# Comparison of divergence-free algorithms for 3D MRI with three-directional velocity encoding

**DOI:** 10.1186/1532-429X-14-S1-W64

**Published:** 2012-02-01

**Authors:** Michael Loecher, Steven Kecskemeti, Patrick Turski, Oliver Wieben

**Affiliations:** 1 Medical Physics, University of Wisconsin Madison, Madison, WI, USA; 2 Radiology, University of Wisconsin Madison, Madison, WI, USA; 3 Physics, University of Wisconsin Madison, Madison, WI, USA

## Summary

Two divergence-free algorithms (finite difference and radial basis functions) are evaluated for improving the flow visualization quality of PC-VIPR 4D flow data. Both methods improve streamline length, with the finite difference method performing slightly better.

## Background

The velocity-to-noise ratio in 4D MR Flow acquisitions can suffer, particularly for low velocity segments, because the velocity encoding settings are adjusted to the highest velocities in the volume. Blood is essentially incompressible so vascular velocity fields should be divergence free. Therefore, any remaining divergence in an acquired 3D or 4D MR flow dataset can be assumed to originate from image noise, which can be reduced by imposing divergence free conditions. Several methods have been proposed to reconstruct a divergence free velocity vector field, yet to our knowledge no comparison studies exist to explore their properties in flow MRI data. This study compares two methods in their effectiveness at improving flow visualizations, the finite difference method (FDM)[1] and the radial basis function (RBF)[2] based approach, which differ in their handling of noise and boundary conditions.

## Methods

MR flow data were acquired in a straight tube flow phantom and the internal carotid arteries of a volunteer on a clinical 3T scanner with a 3D radially undersampled acquistion (PC-VIPR)[3]. Time resolved PC-VIPR images were acquired as follows: scan time: 6 min acquired isotropic spatial resolution: 0.68 mm, TR/TE = 8.3/2.9 ms, temporal resolution: 39.5 ms. The FDM algorithm is solved with a fast Fourier solver for the Poisson equation. The RBF algorithm includes a normalized convolution, and is solved using iterative least squares. The quality of the resulting flow fields was quantified by average streamline lengths and streamline counts that extended from the emitter plane to a distal portion in the flow phantom or vessel. Data fidelity was measured by comparing flow values pre and post processing in the emitter and analysis planes.

## Results

Percentage of streamlines traveling 10cm in the flow phantom: original dataset = 9.2%, FDM = 59.4%, RBF = 40.4%. Average length of streamlines in the flow phantom: original dataset = 6.29cm, FDM = 10.17cm, RBF = 8.10cm. Percentage of streamlines traveling approx. 6 cm along the internal carotid artery: original dataset = 7.4%, FDM = 26.2%, RBF = 12.5%. Average length of streamlines in volunteer: original dataset = 4.41cm, FDM = 5.67cm, RBF = 5.44cm. Variation in flow values from uncorrected data: FDM = 0.1±5.9%, RBF = by 0.6±0.4%. Time to compute: FDM = ~2 min, RBF = ~60 min.

## Conclusions

Both methods, FDM and RBF based, show a significant increase in the streamline length and the number of streamlines that reach a distal portion of the vessel. In our comparisons, the FDM algorithm performed better and was computed significantly faster than the iterative RBF method, whereas the latter introduced fewer variations in flow values. We believe that divergence free methods could become an important component of 4D-MRI flow reconstructions to improve data consistency and visualization.

## Funding

NIH grant R01HL072260.

**Figure 1:**
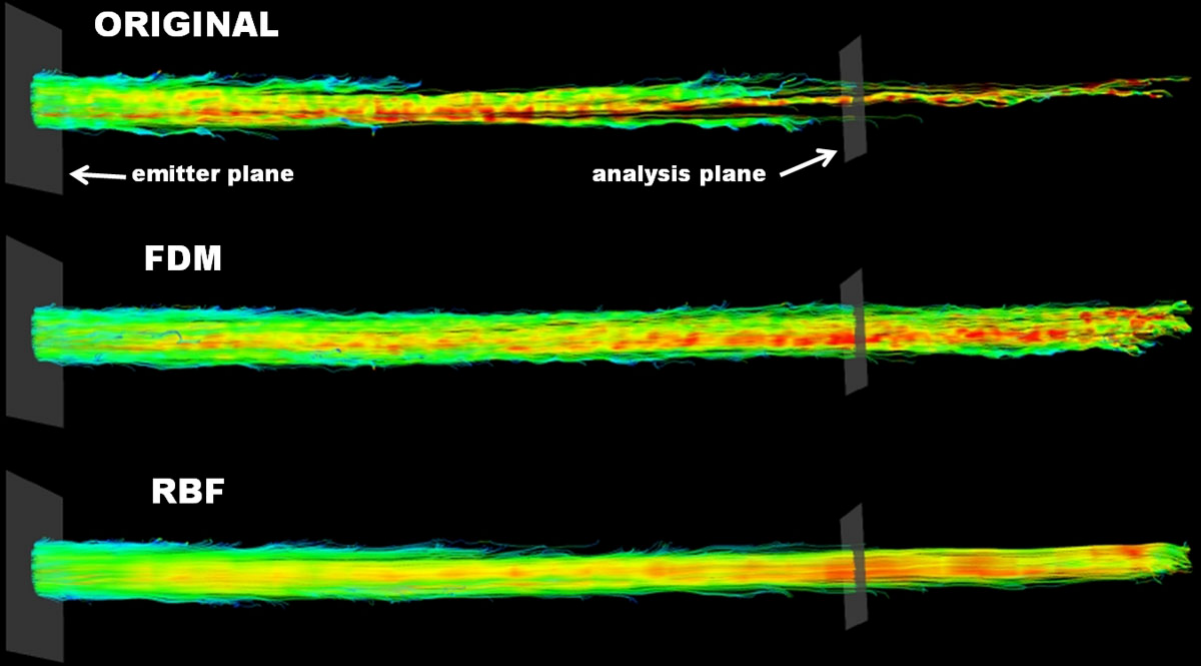
Streamlines through a straight tube flow phantom for the original dataset and the two divergence free correction algorithms. The analysis plane is 10 cm distal from the emitting plane.

**Figure 2:**
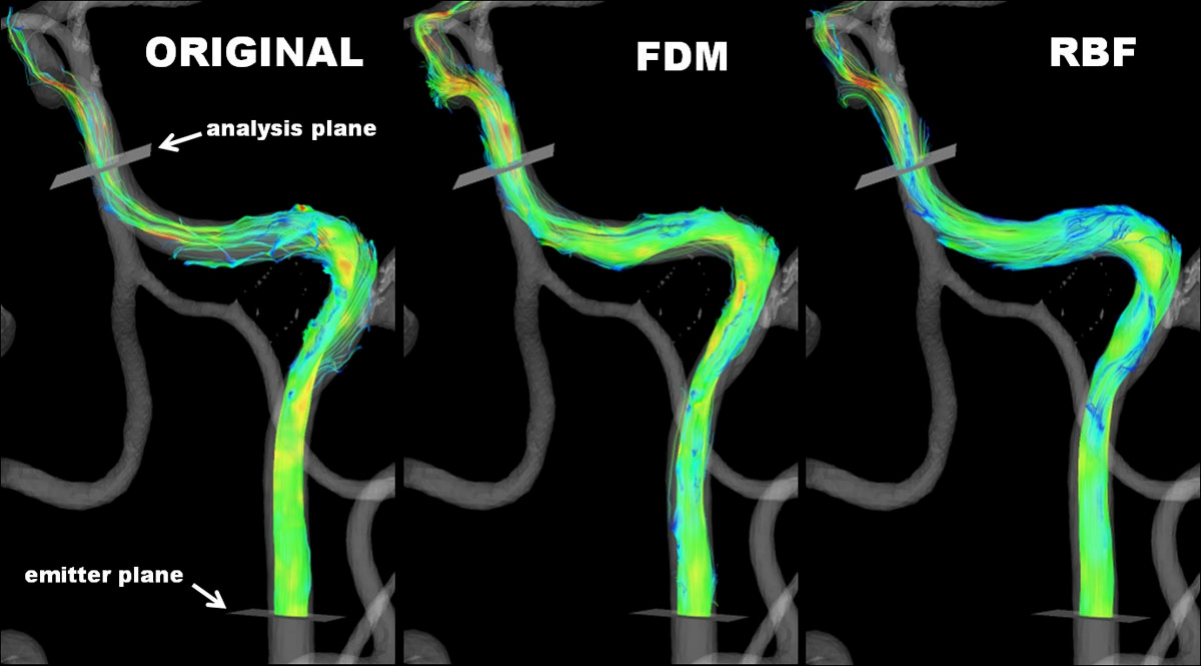
Streamlines through the left internal carotid artery of a volunteer. Streamlines start approximately 1 cm distal to the carotid bifurcation and the analysis plane is placed 6 cm further distal along the artery.

## References

[B1] Song SM JMRI 1993 587 96 10.1002/jmri.1880030407 8347951

[B2] Busch J ISMRM 2011 #1201

[B3] Johnson KM MRM 2008 1329 36

